# Not-in-My-Backyard: Legislation Requirements and Economic Analysis for Developing Underground Wastewater Treatment Plant in China

**DOI:** 10.3390/ijerph15112339

**Published:** 2018-10-23

**Authors:** Meishu Wang, Hui Gong

**Affiliations:** 1 School of Law, East China Normal University, Shanghai 200062, China; mswang@law.ecnu.edu.cn; 2 State Key Joint Laboratory of Environment Simulation and Pollution Control, School of Environment, Tsinghua University, Beijing 100084, China

**Keywords:** underground, wastewater treatment plant, not-in-my-backyard, differences-in-difference, legislation

## Abstract

Underground wastewater treatment plants (WWTPs) have achieved fast development in China in recent years. Due to the remarkable differences between underground and conventional aboveground construction mode, legislation including technical specifications and regulations for underground WWTPs, which was revealed in vacancy, should be issued in time to promote its development. It is also expected to avoid not-in-my-backyard sentiment by decreasing negative effects of WWTPs via construction in sealed underground space. This research took Beijing city as case study to investigate the impacts of WWTPs on nearby community from the perspective of housing price quantitatively. Differences-in-Difference (DID) model result indicates that WWTPs inhibited nearby housing price increases, leading to huge financial losses. The closer are the houses and WWTPs, the severer were the inhibition effects, indicating the relationship between environmental quality and property price. During 2016–2017, the deteriorated estate value surrounding the investigated WWTPs in Beijing was estimated as high as 32.53 billion RMB, much higher than their construction cost of about 4.38 billion RMB. Transformation from grey to green by underground construction was expected to avoid these huge value distortions, while providing alternative to enhance WWTPs with various social functions for public services. This research demonstrates the high social requirements in highly developed cities to promote fast development of underground WWTPs in China.

## 1. Introduction

China’s wastewater treatment industry experienced a super high-speed construction period during the 10th Five-Year Plan (2001–2005) and 11th Five-Year Plan (2006–2010). The number of wastewater treatment plants (WWTPs) in China increased from 481 in 2000 to 3717 in 2014 with wastewater treatment capacity increasing from 22 million t/day to 157 million t/day, accordingly [1,2]. When these WWTPs were planned and built, most were located in the suburbs due to sanitary considerations. However, as urban area has continuously extended in the past decade as a result of fast urbanization process in China, many WWTPs are now surrounded by highly developed urban regions with high population density. WWTPs are usually viewed as “grey” infrastructure since they generate secondary pollutants (e.g., excess sludge, smelly gas and noises) and cause negative impacts on surrounding environment. Thus, “not-in-my-backyard” sentiment is common in many cities with increased complaints for WWTPs, which conflicts with the goals of ecological civilization construction in China [3]. Even though the negative environmental impacts of WWTPs can be reduced by well performed operation and management, their overall intrinsic impacts are still negative. Accordingly, WWTPs industry requires paradigm shift with attention on: (1) how to transform grey infrastructure into ecological green infrastructure; (2) how to change the traditional design philosophy by system synergy and coexistence; (3) how to transform WWTPs from “negative assets” into “positive assets”, which improve value of surrounding community instead of undermining them; and (4) how to satisfy the sustainable requirements including resource recycling, energy conservation and environment friendly [4,5,6].

Underground WWTPs have achieved fast development in China in recent years due to the advantages of eliminating negative environment impacts by constructing treatment facilities in sealed underground space. Sometimes, people do not even notice the existence of the underground WWTPs, which greatly help to avoid “not-in-my-backyard” sentiment. In March 2018, as many as 29 underground WWTPs are in operation in China with additional 20 WWTPs in construction with total treatment capacity over 4 million ton/day, indicating China has constructed the largest underground plant scale in the world. However, two challenges still exist: (1) the shortage of legislation to promote and standardize underground WWTPs planning and construction; and (2) the shortage of economic analysis for value enhancement caused by eliminating negative environment impacts via underground construction. Considering the increased construction cost due to excavating underground space for utilization, economic analysis of underground space utilization is a key parameter for the adoption of such kind of infrastructure over traditional surface solutions and quantitative analysis is useful to evaluate the benefits of underground WWTPs option [7]. 

The research concerning the effects of infrastructures such as WWTPs on surrounding community is limited. Hayes reviewed the methods to evaluate odor impact on communities as well as community response, pointing out analysis of odor impact on communities was at times disjointed [8]. Tzipi measured externalities of waste transfer stations in Israel using hedonic pricing, which were associated with local disamenities experienced by residents living in close proximity, including noise, odor, litter, vermin, visual intrusion and other perceived discomfort [9]. Doron estimated the external effects from aggregate quarries which were in close proximity to population centers by the hedonic pricing method, revealing a decrease of roughly 8.6% in property prices [10]. Hayes implemented a survey investigating the relationships among odor impact, wellbeing, odor observation, and perception, indicating that the odor impact caused by WWTPs was encountered mostly within the 0–1 km range of the plant [11]. Similar research about other public infrastructures in fields such as transportation, business and education (e.g., bus station, large supermarket and schools) are also reported in the literature [12,13,14]. However, no quantitative evaluation of negative impacts of WWTP infrastructures has been reported yet. Moreover, quantitative methods with credible data for evaluation were also in shortage. This research proposed difference-in-difference (DID) method to evaluate the effects of WWTPs on pricing of surrounding houses. The deteriorated value caused by WWTPs could be considered as those could be saved and protected by underground WWTPs. DID method treats the issue of new regulations and policies as objective factors of the “natural experiment” outside of the system [15,16]. DID method could control influences of other factors on the research object, thus separating the real effects of target factor. It could overcome shortage of traditional difference method which could not recognize effects of target factor accurately. DID method is widely used in quantitative evaluation on implementation effects of public policies in econometrics. 

In this study, we reviewed the fast development of underground WWTPs in China. Then, legislation comparison of conventional and underground WWTPs was performed to analyze the legislation requirements to promote underground WWTPs in China. Moreover, negative impacts of WWTPs on nearby community was evaluated by DID model. We took Beijing city as case study to investigate the impacts of WWTPs on nearby community from the perspective of housing price quantitatively, evaluating the internal economic motivation of underground WWTP developments, especially in megacities.

## 2. Current Development of Underground WWTP in China

As early as the 1980s, underground wastewater treatment plant was built to resist cold climate such as VEAS WWTP in Oslo of Norway [17]. Due to limited land resources caused by highly developed urbanization and population increase, underground wastewater treatment plants started to demonstrate advantages of more efficient space utilization with its wide construction all over the world, especially in Europe and Asia. There are underground plants reported in the Netherlands, Norway, Sweden, France, Finland, Japan, Korea, Malaysia, etc.

In China, construction of underground plants dated back to the 1990s when Stanley WWTP in Hong Kong was built in caves to save land resources. Until recently, rapid development for underground plants construction has occurred, promoted by rapid increase of economy and population in China. One important case is Buji WWTP in Shenzhen city of China, which chose Anaerobic/Anoxic/Oxic (A/A/O) process with design scale as large as 200,000 m^3^/day. The case was landmark by demonstrating stable operation in large-scale and all-underground plant since its commissioning in 2011 with National First Class—A effluent discharge standard.

During the 12th Five-Year Plan (2011–2015), more underground WWTPs were constructed in China: 29 plants in operation with 3.19 million t/day treatment capacity. As shown in Table 1, although the number of underground plants only account for 2.1% of conventional plants during 12th Five-Year Plan, the treatment capacity and investment were as high as 6.9% and 13.9%, respectively, indicating underground WWTP is an important part of wastewater treatment industry. 

Based on our investigation, in March 2018, besides 29 underground WWTPs in operation there are an additional 20 WWTPs under construction with total treatment capacity over 4 million t/day in China. It is expected that the amount of underground WWTPs will continue to increase with market value more than 50 billion RMB. Now, underground WWTPs are built in both megacities (such as Beijing, Guangzhou and Shenzhen) and provinces with limited land resources (Guizhou, Yunnan and Sichuan) in China.

## 3. Legislation Comparison of Conventional and Underground WWTPs

Technical standards and specifications for conventional WWTPs are mature in terms of planning, design, construction and operation (Table 2). In comparison, as a new-born construction pattern, the legal requirements and documents for underground WWTP are very limited, but are needed to promote its fast developments [18,19]. Due to the remarkable differences between underground and conventional aboveground WWTPs in terms of process selection, building structure, vertical design, operation and maintenance, the requirements for conventional aboveground WWTPs cannot be directly used for underground plants [20]. For example, both underground and aboveground WWTPs apply for discharge standards and limits for municipal wastewater treatment plants (GB 18918-2002) since effluent water quality must be guaranteed in all cases. However, conventional surface WWTPs are suitable for noise standards and limits for industrial enterprises (GB 50348-2004), while underground WWTPs are not, which should consider their special sealed condition. In addition, while most requirements for construction and design are suitable for conventional WWTPs, additional requirements for underground WWTPs are needed due to safety consideration. In addition, valuable experiences should also be summarized and spread in the form of legal technical specifications and regulations.

There are national plans and legislation for underground space utilization for transportation and commercial development, e.g., subway and underground supermarket. However, no specific legal policy has been issued for underground WWTPs. Considering its fast development, legislation should be enhanced in following perspectives. 

Technical specifications and regulations for process design of underground WWTPs should be issued. Process designs are mature for conventional projects with consideration of discharge limits and wastewater characteristics. However, technical specifications and regulations for underground project are absent. One great difference is the process with small footprint is preferred for underground WWTPs since it could save considerable construction cost. Currently, more than half of underground projects have chosen modified AAO process, while about one quarter membrane bio-reactor (MBR) process. Most small-scale underground projects prefer moving bed biofilm reactor (MBBR) process. In contrast, processes including oxidation ditch and cyclic activated sludge system (CASS), whose footprints are large, are seldom applied. The specific land occupation (area per ton wastewater treated) for underground plant is 0.30–0.41 m^2^/t and 0.42–0.60 m^2^/t for large and small scale, respectively, both being smaller than conventional aboveground projects (0.5–1.5 m^2^/t).

Technical regulations for odor control and environmental evaluation should also be specifically defined for underground plants. Since they are built in sealed underground space, all odor should be and could easily be collected and treated, which is quite different from the open design of aboveground project with disorderly emission. In addition, as they are visible, the negative feeling with less environmental impacts is achieved, indicating the unnecessary of traditional long distance isolation requirements.

Finally, basic planning principle for underground WWTPs should be emphasized. Not all situations are suitable for underground option. For highly developed regions such as megacities where population load is severe and land resources limited and expensive, underground WWTPs are the market choice. Besides, in environmentally sensitive regions, underground WWTPs are also planned with simultaneous intentions of pollutant control and ecological compensation for rivers.

## 4. The Effects of WWTPs on Surrounding Housing Pricing—Case Study in Beijing

### 4.1. Brief Introduction of WWTPs in Beijing

**Figure 1:**
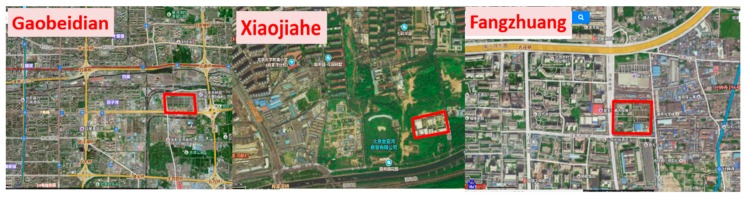
Aerial images of Gaobeidian, Xiajiahe and Fangzhuang WWTPs in Beijing (red line indicates boundary of WWTPs).

Currently, there are seven WWTPs located in District of Haidian, Zhaoyang and Fengtai in Beijing. Here, WWTPs in Beijing could be divided into two types. The first type includes Fangzhuang, Xiaojiahe and Gaobeidian WWTP, which are surrounded by highly developed communities. Most of the WWTPs were constructed at least 15 years ago (Table 3). When they were constructed, they were located in remote regions of Beijing. However, after years of rapid urbanization, the locations of these WWTPs are no longer “remote areas” and many are surrounded by dense residential areas (Figure 1).

The second type includes the Qinghe, Beixiaohe, Jiuxianqiao, Xiao Hongmen and Beiyuan WWTPs, which were constructed near green parks. For example, Qinghe plant was close to the west of Beijing Olympic Park. In most cases, the occupied lands by parks were much larger than those by WWTPs. The negative effects of WWTPs may be covered by the positive effects of green parks when they are built together. Thus, in this study, only the three solo built WWTPs—Gaobeidian, Xiajiahe and Fangzhuang—were selected to evaluate their impacts on surrounding housing prices.

### 4.2. Acquisition and Processing of Housing Price Data

Housing price data were acquired from the open information on Internet. The prices of one day in 2016 and 2017 were obtained for analysis. Each datum contained housing price per unit area, community name, construction year of the community, administrative region, the location, etc. Based on location information (longitude and latitude), the straight distances between houses and various WWTPs were calculated and used to evaluate the effective scope of impacts caused by WWTPs.

### 4.3. Difference-in-Difference Method

In DID model, the influences of background factors were obtained through difference of the control group, and then the collaborative influences of background factors combined with target factors were obtained through differences of the target group. Accordingly, the specific influences of target factors were obtained through the double differences between the two groups, namely DID method. In this study, DID method was used to evaluate the impacts of WWTPs on surrounding housing price.

The housing price Price(*P_R,T_*) is described as follows:

Price (*P_R,T_*) = *β*_0_ + *β*_1_*D_RT_* + *β*_2_*R* + *β*_3_*T* + *εRT* (1)

where *R* is the region and its value is 1 for the target group (surrounding regions of WWTPs) and 0 for the control group. *T* is time, whose value is 0 for 2016 and 1 for 2017. *D_RT_*= *R* × *T*, describing the cross term of regional dummy variable and time dummy variable. *ε* is the random error term and obeys the standard normal distribution. *β*_1_ is a parameter that is expected to be acquired and indicates the influenced value on housing prices. The numerical value of *β*_1_ reflects influences of WWTPs on increase of housing price in their surrounding regions. If it is positive, it indicates that WWTPs promote the increase of housing price in surrounding regions. If it is negative, WWTPs inhibit the increase of housing price.

For control group in 2016:

Price(*P*_0,0_) = *β*_0_ (2)

For control group in 2017:

Price(*P*_0,1_) = *β*_0_ + *β*_3_ (3)

For target group in 2016:

Price(*P*_1,0_) = *β*_0_ + *β*_2_ (4)

For target group in 2017:

Price(*P*_1,1_) = *β*_0_ + *β*_1_ + *β*_2_ + *β*_3_ (5)

According to Equation (1)–(5), *β*_1_ could be calculated as follows:

*β*_1_ = [Price(*P*_1,1_) − Price(*P*_1,0_)] − [Price(*P*_0,1_) − Price(*P*_0,0_)] (6)

The basic hypothesis of DID method requires the control group is not impacted by target factors and shows identical development trend (parallel trend hypothesis) with the target group when influences of target factors are eliminated. In this study, there were two requirements for the target and control group. Firstly, the housing price of control group should not be impacted by WWTPs. In other words, the houses in control group should be located in regions beyond scope influenced by WWTPs. It was hypothesized that the scope influenced by WWTPs is a round radiation with effective radius of 1 km [11], which was used to define scope of target group. Based on the distance from WWTP, the target group was further divided as Subgroup 1 and Subgroup 2 with distances of 0–0.5 and 0.5–1 km, respectively (Table 4). Secondly, the control group should have the same local characteristics with the target group except the distance to WWTPs. The annular region with a width of 1 km outside the effective radius of WWTPs was chosen as the control group in this research. The housing price was influenced by both internal characteristics of house (e.g., decoration quality) and external characteristics of local situations (e.g., transportation and supporting facilities). The houses with various internal characteristics were considered randomly located around the city. To avoid variation of external characteristics, the round target group and the annular control group were located in the same location with a radius of 2 km. Considering the very large area of Beijing city (30 km × 30 km of Fifth Ring Road in Beijing), the houses in this local atmosphere were considered with the same external characteristics, which accorded with the parallel trend hypothesis.

### 4.4. DID Results: Inhibited Housing Price Increase by Surrounding WWTP

The evaluated WWTPs (Fangzhuang, Xiaojiahe and Gaobeidian) produced direct negative impacts on surrounding environment and the housing price was inhibited significantly. In addition, the negative impacts intensified as approaching to the plant. As shown in Table 4, the specific damaged value for Subgroup 1 (within 0.5 km of WWTP) and Subgroup 2 (0.5–1 km of WWTP) was 4697.6 and 2776.1 yuan RMB/m^2^, respectively.

The overall inhibition effects of the Fangzhuang WWTP on surrounding housing price were further analyzed. We assumed the situation that the land around Fangzhuang was fully developed. According to *Planning and Design Specification for Residential Areas in Cities (GB50180-93)*, it was regulated that four major land types, residential area, industrial area, road/square area and greenbelt area, should account for 20–32%, 15–25%, 8–15% and 8–15% of the total construction land area, respectively. Here, the highest value of 32% for the proportion of residential area was used for calculation. The volume fraction of houses in this community was set as 2.46, equal to the averaging value of the whole Fengtai District where Fangzhuang WWTP is located. The housing value loss caused by negative impacts of WWTP on housing price increase during 2016–2017 was calculated as:

M = M_0.5_ + M_0.5–1_ = [500^2^ × *β*_1_(<0.5) + (1000^2^ − 500^2^) × *β*_1_(0.5–1)] × π × 32% × 2.46 (7)

As shown in Table 5, it could be seen that WWTPs caused loss as much as 32.53 billion RMB. Meanwhile, the values of land resources occupied by WWTPs were calculated. It should be noted that the land values occupied by WWTPs was 143.03 billion yuan (estimated with the same land price of Fangzhuang in 2017), which was significantly higher than the total investment estimation for construction (about 4.38 billion RMB). It was revealed that controlling negative impacts of WWTPs in large cities with high housing price (e.g., Beijing) and utilizing their occupied land resources could achieve remarkable economic benefits. The construction mode of underground WWTPs provided opportunity to make more use of land resources, which was alternatively occupied by conventional aboveground WWTP. Most underground WWTPs have been co-built with green parks in their above space in China.

It should be noted that this research only focused on the housing value variation during 2016–2017. Beijing witnessed an unexpected dramatic increase of house pricing during this time, which may magnify the negative effects of WWTPs. Considering the long-term existence of WWTPs, their total inhibition effects might accumulate and last. It would be more helpful if more data over longer period could be collected to analyze the impacts of WWTP on surrounding house pricing in long term, which could be considered in further research. In addition, there were certain limitations since DID model was based on some hypotheses and simplifications. One important hypothesis was that the houses with various internal characteristics (e.g., size of apartments, number of stories, and decoration quality) were considered randomly located around the city. Statistical evidence was not analyzed due to lack of suitable initial data. Furthermore, all housing price data were collected from open data on the Internet instead of official data and their reliability cannot be 100% guaranteed. It is possible that there was false information, which would influence the final quantitative results. For example, people may claim lower sale price on Internet to draw attention while the true prices were actually high. All these factors were overlooked in this paper.

## 5. Perspectives of Underground Wastewater Treatment Plant in China

WWTPs should not only pursue energy saving and resource recycling by technological improvements [21,22,23], but also pay more attention to the relationship between WWTPs and surrounding communities by improving their environmental friendliness. With continuous expansion of urban boundaries, more and more WWTPs, for example the case in Beijing, were surrounded by highly developed communities. Due to the continuous urbanization and scarcity of land resources, both the land area occupied by WWTPs and surrounding WWTPs were no longer cheap. Analysis based on housing price in this research provided some evidence that reflected the contradiction and value distortion of traditional WWTPs in highly developed cities.

Demolition and reconstruction is one possible solution. Taking down the old one in city center and building a new one out of the city could lessen land shortage and avoid not-in-my-backyard sentiment. However, this option suggests abundant sewage generated in urban area would have to be transported a long distance, which would increase investment cost (e.g., pipeline system) and difficulties for resource reused [24].

**Figure 2:**
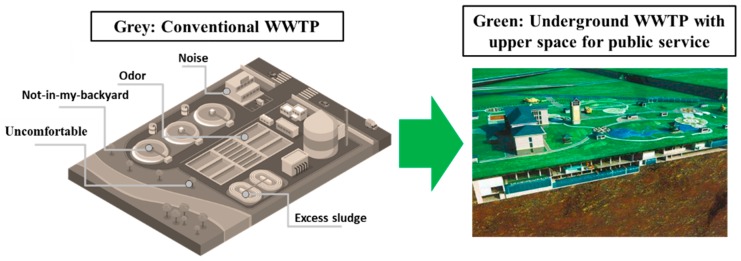
Concept of transformation from grey to green by underground construction.

Transformation from grey to green by underground construction is a new method worthy of discussion. Within boundary of WWTPs, there is not only water, which is the core element to support ecosystem, but also land, which is fundamental for development of public services and functions (e.g., entertainment and education). As shown in Figure 2, transition from grey to green by underground construction provides a new solution, which make WWTPs no longer a single wastewater treatment facility that everyone avoids but a complex with function of both water treatment and public services. For example, underground WWTPs become a new green infrastructure that could realize various social functions simultaneously, including public education, ecological civilization, and cultural entertainment. It should be noted that the combination of WWTPs and green facilities should be carefully designed fulfilling social requirements of nearby communities instead of simple adjacent construction [25]. The microbiological impact and risk when reclaimed wastewater is used for recreational parks should also be investigated [26]. The increasing development of underground WWTPs in China reflects the higher social requirements for public infrastructures in highly developed cities.

## 6. Conclusions

Fast development of underground WWTPs is happening in China, especially in megacities with limited land resources and high population. Due to the remarkable differences between underground and conventional aboveground modes in terms of process selection, building structure, vertical design, operation and maintenance, legislation including technical specifications and regulations, which is currently absent, should be issued as soon as possible to promote development of underground WWTPs. Underground WWTPs protect surrounding community value with less not-in-my-backyard effects. Based on housing price data, Differences-in-Difference (DID) model result indicates that WWTPs inhibited nearby housing price increases, leading to huge financial losses. The closer are the houses and WWTPs, the severer are the inhibition effects. Both the value of land occupied by WWTPs and the real estate value loss caused by WWTPs were far beyond the investment for WWTPs construction itself. During 2016–2017, the deteriorated estate value surrounding three investigated WWTPs was estimated as high as 32.53 billion RMB, much higher (about 7.5 times) than their construction cost of about 4.38 billion RMB. Transformation from grey to green by underground construction could avoid huge value distortions of aboveground WWTPs and provide alternative to enhance WWTPs with various social functions for public services.

## Figures and Tables

**Table 1:** Comparison of new built conventional and underground WWTPs during 12th Five-Year Plan (2011–2015) in China.

Type of WWTPs	Conventional	Underground
WWTPs number	1370	29 (2.1%)
Treatment capacity (million t/day)	46	3.19 (6.9%)
Investment (billion RMB)	92	12.8 (13.9%)

**Table 2:** Standardized documents including technical standards and specifications for conventional WWTPs.

Category	Document Titles
Technical specification and regulations for process design	Code for outdoor drainage design (GB50014-2006, 2016 version)
	Technical specification for constructed wetland (HJ 2005)
	Technical specification for coagulation and flocculation wastewater treatment (HJ 2006)
	Technical specification for biological contact oxidation wastewater treatment (HJ 2009)
	Technical specification for the membrane bio-reactor wastewater treatment (HJ 2010)
	Technical specification for biological filter wastewater treatment (HJ 2014)
	Technical specification for anaerobic–anaerobic–aerobic activated sludge wastewater treatment (HJ 576)
	Technical specification for batch activated sludge process wastewater treatment (HJ 577)
	Technical specification for oxidation ditch wastewater treatment (578)
	Technical specification for membrane separation wastewater treatment (HJ 579)
	Technical regulations for sludge treatment of municipal wastewater treatment plants (CJJ 131-2009)
	Technical regulations for odor treatment of municipal wastewater treatment plants (CJJ/T 243-2016)
Requirements for construction and design	Code for fire safety of building design (GB50016-2014)
	Specification for building structures Load design (GB 50009)
	Technical specifications for retaining and protection of foundation excavation (JGJ 120-2012)
	Specification for structural design of water supply and drainage (GB 50069)
	Specification for heating ventilation and air conditioning of industrial buildings (GB 50019-2015)
Discharge standard and limits	Discharge standards and limits for municipal wastewater treatment plants (GB 18918-2002)
	Noise standards and limits for industrial enterprises (GB 50348-2004)

**Table 3:** Information of WWTPs in Beijing.

Location (District)	WWTP Name	Treatment Process	Operation Year	Treatment Capacity (×10^4^ t/day)	Land Occupy (×10^4^ m^2^)
Fengtai	Fangzhuang	A/A/O	1995	4	4.9
Haidian	Xiaojiahe	A/A/O	2003	2	3.4
Chaoyang	Gaobeidian	A/O	1999	100	67.9

**Table 4:** Comparison of *β*_1_ for WWTPs in Beijing.

*β*_1_ (yuan RMB/m^2^)	Fangzhuang ^a^	Xiaojiahe ^a^	Gaobidian WWTP ^b^
Subgroup 1 (<0.5 km)	−4697.6 *	ND ^c^	ND ^c^
Subgroup 2 (0.5–1 km)	−2776.1 **	−2989.7	−4208.5 *

^a^ The control group was annular scope within distance of 1–2 km of WWTP. ^b^ Considering the land scope occupied by Gaobeidian WWTP was very large due to its huge treatment capacity, the distances for Subgroup 1, Subgroup 2 and control group were adjusted to 0.5–1 km, 1–2 km and 2–3 km. ^c^ ND (No data) indicated no housing data were collected in the region. ** and * indicate statistically significant at 5% and 10% level, respectively.

**Table 5:** Comparison of deteriorated estate value, land price and construction investment for WWTPs in Beijing.

WWTP Name	Deteriorated Estate Value by WWTPs (billion RMB, 2016–2017)	Value of Land Resources Occupied by WWTPs (billion RMB)	Investment Cost for Construction ^a^ (billion RMB)
Fangzhuang	7.47	9.22	0.12
Xiaojiahe	5.55	6.37	0.06
Gaobeidian	19.51	127.44	3.0
Total (billion RMB)	32.53	143.03	4.38

^a^ The investment was estimated based on investment of 3000 RMB/ton for WWTPs construction.
